# Short-term and Potentially Long-term Negative Impacts of COVID-19 in Sub-Saharan Africa: Evidence from the Africa Research, Implementation Science, and Education Network Rapid Monitoring Survey

**DOI:** 10.4269/ajtmh.21-0617

**Published:** 2021-06-23

**Authors:** Davidson H. Hamer

**Affiliations:** Department of Global Health, Boston University School of Public Health, Boston, Massachusetts;; Section of Infectious Disease, Department of Medicine, Boston University School of Medicine, Boston, Massachusetts;; National Emerging Infectious Disease Laboratories, Boston, Massachusetts

As of early May 2021, there have been more than 4.67 million cases of coronavirus disease 2019 (COVID-19) and nearly 126,000 deaths reported in Africa.^1^ These figures are likely substantial underestimates because of limited testing capacity and national surveillance programs in many parts of the continent. Like much of the world, Africa experienced a second wave of COVID-19, which appeared more severe than the first wave, that started in late 2020 and continued into 2021.^2^ Beyond its impact on health systems and direct consequences of infection for afflicted individuals, there may be more widespread indirect socioeconomic adverse impacts of COVID-19.

To understand COVID-19 knowledge, attitudes, and practices and their impacts on health, nutrition, and education, the Africa Research, Implementation Science, and Education (ARISE) Network conducted a telephone survey at six sites (one urban and one rural in each country) in Burkina Faso, Ethiopia, and Nigeria between July and November 2020.^3^ Three different groups, adolescents, adults, and healthcare workers, were surveyed using a mobile telephone survey platform. Results of these surveys are reported in this supplement to the *American Journal of Tropical Medicine and Hygiene*.

There have been wide variations in the levels of knowledge of COVID-19 symptoms and modes of transmission among adolescents, adults, and healthcare workers. Although the level of knowledge of common symptoms and the perception of personal risk of severe acute respiratory syndrome coronavirus 2 (SARS-CoV-2) infection were relatively low among adolescents, prevention approaches (e.g., face mask use and physical distancing) were commonly practiced.^4^ In contrast, adults in the general population and healthcare providers generally had better levels of COVID-19 knowledge, although nurses had more limited knowledge than physicians.^5^^,^^6^ Notably, healthcare workers commonly reported the use of prevention measures, especially if they were treating COVID-19 patients, and 74% described self-perceived social stigma; fortunately, the rates of psychological distress, anxiety, and depression were low.^6^ Despite potentially negative effects on adult and adolescent mental health, the surveyed population did not appear to have been greatly impacted, with only 21% of adults having mild psychological distress and very few adolescents experiencing anxiety and depression.^4^^,^^5^

These surveys revealed many worrisome findings. The majority of adolescents had greatly decreased communication with family and friends, were no longer attending school because of closures, could not continue their education during the pandemic, and anticipated not being able to catch-up with what they missed after the pandemic.^4^ Food insecurity was another major challenge, with decreased consumption of a range of staple foods across all countries in both rural and urban locations. Increases in prices of staples, legumes, vegetables, fruit, and animal-source foods, decreased crop production, and unemployment all contributed to many survey respondents describing reductions in food intake and dietary diversity.^7^ The impact of food insecurity was greater in urban areas than in rural areas.

In addition to the adverse effects on education and food security, the survey found partial or complete interruptions in the delivery of basic maternal and child healthcare services, including pediatric immunization services, vitamin A supplementation, and access to preventive nutrition services and malnutrition treatment.^8^ There were similar difficulties for women of reproductive age, with approximately one-quarter experiencing challenges accessing family planning services, antenatal care, and iron and folic acid supplementation. There were also problems, albeit more limited, accessing human immunodeficiency virus treatment (18%) and urgent surgical procedures (14%).

Although these studies had several limitations, including differences in consent processes between countries (in-person informed consent was required in Ethiopia but not elsewhere), a lack of generalizability beyond the study communities, variations in sampling frameworks across the study locations, and the availability of completed surveys only from willing individuals with mobile telephones, there were also several strengths. Diverse samples of the general population in rural and urban areas were recruited across three countries, there was a focus on the impact on adolescent health and education, and data were disaggregated by age and sex. Additionally, mobile telephone surveys represent a potentially low-cost strategy for data collection in contrast to in-person household surveys.

This three-country telephone survey revealed varying levels of knowledge of COVID-19 symptoms and how this disease is transmitted, although most adults and healthcare workers reported using effective preventive measures. However, the more concerning findings were related to the negative adverse effects of the COVID-19 pandemic on routine health services, access to education for adolescents, and food security and dietary diversity. The short-term impacts of undernutrition and impaired access to basic healthcare for children, adolescents, and adults are likely to be substantial. These problems are likely to be further exacerbated by the ongoing second wave of the pandemic, which appears to be more severe in sub-Saharan Africa. The combination of food insecurity leading to undernutrition and major challenges regarding completing education may have long-term negative effects on adolescents and younger children who may be greatly impacted by food insecurity and reduced dietary diversity in terms of their future health, nutritional status, and potential for economic development.^9^
